# *CDH23* Methylation Status and Presbycusis Risk in Elderly Women

**DOI:** 10.3389/fnagi.2018.00241

**Published:** 2018-08-07

**Authors:** Amal Bouzid, Ibtihel Smeti, Amine Chakroun, Salma Loukil, Abdullah Ahmed Gibriel, Mhamed Grati, Abdelmonem Ghorbel, Saber Masmoudi

**Affiliations:** ^1^Laboratory of Molecular and Cellular Screening Processes, Center of Biotechnology of Sfax, Sfax, Tunisia; ^2^Department of Otorhinolaryngology, Habib Bourguiba Teaching Hospital, University of Sfax, Sfax, Tunisia; ^3^Unité de Recherche Surdité et Cancer du Cavum, UR12ES21, Faculté de Médecine, Université de Sfax, Sfax, Tunisia; ^4^Biochemistry and Molecular Biology Department, Faculty of Pharmacy, The British University in Egypt (BUE), Cairo, Egypt; ^5^Department of Otolaryngology, University of Miami, Miller School of Medicine, Miami, FL, United States

**Keywords:** presbycusis, *CDH23*, DNA methylation, CpG sites, quantitative methylation-specific PCR

## Abstract

**Introduction**: Presbycusis, an age-related hearing impairment (ARHI) disease, is the most common cause for HI in adults worldwide. One of the best candidate genes for ARHI susceptibility is Cadherin 23 (*CDH23*) which encodes stereocilia tip-links of the inner ear sensory hair cell. Although alterations in the methylation status of CpG dinucleotides across various genes were reported to be associated with HI, methylation changes in *CDH23* gene have not been reported previously.

**Objectives**: This study aimed at investigating whether DNA methylation level of *CDH23* gene at intragenic CpG island overlapping an exonic-intronic region at position chr10:73565570-73565827 (GRCh37/hg19) could be risk factor associated with ARHI.

**Materials and Methods**: We screened for methylation changes in this particular position for *CDH23* gene in 50 blood samples of elderly women affected with presbycusis and healthy control cohort. Methylation of CpG sites were assessed using Quantitative methylation-specific PCR (qMSP) following sodium bisulfite DNA conversion chemistry. Methylation levels were normalized against *TSH2B* reference gene.

**Results**: DNA methylation analysis for the common CpG islands in *CDH23* gene revealed 3.27-folds significant increase (*p* < 0.0001) in methylation profile for ARHI women as compared to healthy controls with an elevated risk odds ratio (OR) of 2.219 [95% CI 1.071–4.597].

**Conclusion**: Our study is the first of its kind to prove that higher CpG site methylation levels in *CDH23* gene are likely to be associated with ARHI.

## Introduction

Age-related hearing impairment (ARHI), named also presbycusis, is the most common form of sensory disability in elderly individuals. The World Health Organization (WHO) estimated that 466 million people worldwide are disabled with HI^1^. Presbycusis is characterized by bilateral, symmetrical and non-conductive type of hearing defects. It is associated with negative consequences in communication and social life quality. The causative molecular pathogenesis for ARHI is still unknown (Tu and Friedman, 2018). Therefore, investigating molecular pathogenic mechanisms underlying presbycusis is very crucial prior to development of cost-effective therapeutic strategies.

Cadherin 23 (*CDH23*), also known as otocadherin, is part of the cadherin super-family of calcium-dependent cell-surface adhesion proteins. *CDH23* encompasses 69 exons (NM_022124) and encodes a 3354 amino acid protein (NP_071407.4) with 27 extracellular cadherin domains, a single trans-membrane domain and a short cytoplasmic domain. *CDH23* was reported to play a key role in lateral (Lagziel et al., 2005) and stereociliary tip-links (Siemens et al., 2004) of the inner ear sensory hair cells that control hearing process. Tip-links are extracellular filaments that are the “gate cables” for opening mechano-transduction channels which transduces mechanical forces arising from sound waves and head movement, allowing hearing and balance to occur (Pickles et al., 1984). Thus, mutations in *CDH23* gene are associated with distinct sensory impairments (Siemens et al., 2004). Missense mutations in *CDH23* gene (NM_022124) have been associated with severe to profound non-syndromic pre-lingual hearing loss *DFNB12* (McHugh and Friedman, 2006). Meanwhile, deletions, nonsense or frame-shift mutations were reported to cause Usher syndrome (*USH1D*). This clearly indicates that mutations classes are closely related to the phenotype (Petit et al., 2001).

Qualitative and quantitative linkage studies on mice linked ARHI genetic susceptibility to *Ahl* locus on chromosome 10. The *Ahl* allele, also known as *Cdh23*^753A^, is a recessive synonymous single nucleotide polymorphism (SNP; c.753G>A) in exon 7 of *Cdh23* gene that was significantly associated with ARHI in common inbred mouse strains (Johnson et al., 2000). The hypomorphic *Cdh23* *Ahl* allele was reported to be common in at least *C57BL/6, BALB*, and *129S6* ARHI models and to cause in-frame skipping of exon 7 (Noben-Trauth et al., 2003). Although the *Ahl* allele of *Cdh23* gene was proven to be linked with ARHI in mice, there is no reported evidence for the implication of *CDH23* intron-7 SNP in ARHI in humans (Hwang et al., 2012).

Epigenetic hearing modifications in elderly individuals were responsible for impaired hearing in both ARHI and syndromic cases (Provenzano and Domann, 2007). Interestingly, recent investigations demonstrated that aberrant methylation of HI genes in children could lead to the development of ARHI. Hypermethylation of *GJB2* promoter region in the mimetic aging rat cochlea was associated with down regulation of connexin 26 that resulted in the development of presbycusis (Wu et al., 2014). Moreover, hypermethylation of CpG sites in the promoter region of *SLC26A4* (*DFNB4*) and *P2RX2* (*DFNA41*) genes resulted in increased risk for presbycusis in men (Xu et al., 2017), and a down-expression in elderly women with presbycusis (Bouzid et al., 2018), respectively. The aim of the present study was to correlate *CDH23* methylation profiles with ARHI phenotype characteristics to investigate possible use of *CDH23* gene as a novel epigenetic marker in ARHI. This could pave the way towards understanding complex pathogenic mechanisms underlying ARHI.

## Materials and Methods

### Subjects and Observed Phenotype

The present study was approved by ethics committee of the University Hospital of Sfax (Tunisia). Informed and written consent was obtained from all individual participated in this study. Clinical, biological and pure tone audiometric data were collected from 50 unrelated age-matched subjects (25 patients and 25 controls) ranging from 50 years to 75 years.

Selected women were classified into controls and affected groups based on mean bone conduction threshold as a function of age as indicated in our previously published study (Bouzid et al., 2018). Wherein, it was reported that hearing loss thresholds were assessed through 95% confidence interval (CI) for means of bone conduction values across increasing ages (*p* < 0.05). Subjects with hearing loss below the lower limit of the 95% CI were considered as control group (with hearing loss thresholds <20 dB) while subjects with hearing loss above the upper limit of the 95% CI were classified in ARHI group (with hearing loss thresholds ≥20 dB) showed typically presbycusis audiograms with a downgrade into higher frequencies. Individuals having ARHI risk factors such as occupational noise, medication induced ototoxicity, otitis media infection history, hypertension, diabetes, dyslipidemia and family history for hereditary HI were excluded from this study.

### Evaluation of *CDH23* CpG Island Sites

CpG island sequence conservation for *CDH23* gene was evaluated using tools provided by the UCSC Genome Browser interface. Evolutionary conservation in 100 vertebrates is generated using multiz alignment and scored by the phastCons and phyloP programs (Siepel et al., 2005). Scores of evolutionarily constrained elements were estimated using the GERP++ method (Davydov et al., 2010). Classification of *CDH23* CpG-rich regions according to their evolutionary dynamics was examined using parameter-rich evolutionary model and clustering analysis (Cohen et al., 2011). For the *CDH23* CpG island sites, chromatin non-condensed DNaseI Hypersensitivity regions in 125 cell types tracks were assessed from ENCODE project (V3).

### Quantitative Methylation-Specific PCR

The bisulfite conversion method was implemented using innuconvert bisulfite basic kit (AnalytikJena, Jena, Germany) according to the manufacturer’s recommendations. Bisulfite-modified DNA was then used as template for the real-time PCR. The *CDH23* primers, F: 5’-CAAAGTCACAGGAAGTGTGC-3’ and R: 5’-CATTCACATCCAGGACAGTG-3’, were designed to amplify an amplicon size of 137 bp in the CpG island site of the junction exon 54–intron 54 (NM_022124). For endogenous normalization purposes, *TSH2B* promoter primers were used to amplify a highly methylated CG rich region in all somatic cells (Diagenode, Liège, Belgium). Quantitative methylation-specific PCR (qMSP) was performed for both *CDH23* and *TSH2B* genes.

To ensure selectivity of those primers in *CDH23* gene, a positive control genomic methylated bisulfite converted DNA and a negative control genomic unmethylated bisulfite converted DNA (Diagenode, Liège, Belgium) were used. The former control contains methylated cytosine residues to ensure amplification of methylated products. The positive control would also evaluate methylation levels for both ARHI and control samples. The latter contains uracil residues to ensure no amplification for un-methylated DNA.

The PCR reaction mix contained Syber Premix of the Episcope MSP kit (TakaRa, Otsu, Japan), 10 μM of each primer and 100 ng of bisulfite-modified genomic DNA template in a final volume of 10 μL. PCR amplification was performed using CFX96 Real-Time PCR detection system (Bio Rad, Redmond, WA, USA). All samples were run in duplicates for both target and reference genes. The real-time PCR conditions were as follows; begun with an initial denaturation step at 95°C for 30 s, followed by 40 repetitive cycles at 98°C for 5 s, 60°C for 40 s and extension at 72°C for 30 s. Melt curve analysis was performed for all samples following RT-PCR run. The Bio-Rad CFX Manager software (Bio Rad, Redmond, WA, USA) was used to interpret data.

The quantification cycle was defined for each reaction and the ratio of methylated to total amplifiable bisulfite-treated DNA was determined (Bustin et al., 2009). The relative level of methylated DNA for each sample group of patients with presbycusis and controls was carried out using comparative threshold cycle. To evaluate different CpG methylation levels between ARHI patients and controls, the ΔΔCq method was applied using the *TSH2B* as a reference gene (Schmittgen and Livak, 2008).

### Statistical Analysis

Statistical analyses were performed with GraphPad Prism software version 5.0 (La Jolla, CA, USA). Differences in methylation levels between presbycusis patients and controls were compared with Student’s *t*-test. *P*-values ≤0.05 were considered statistically significant. To assess correlation between *CDH23* gene methylation levels and hearing impairment, the two tailed Fisher’s exact test was used. Odds ratio (OR) was explored as a part of association with a 95% CI as an appraisal of precision for this evaluation.

### RNA Extraction and RT-PCR Analysis

Total RNA extraction from peripheral blood of three individuals, with different ages 60, 64 and 70 years old, was performed using PAXgene Blood RNA Kit (PreAnalytiX, Hombrechtikon, Switzerland) according to manufacturer’s instructions. Extraction yield was evaluated with Nanodrop2000 spectrophotometer (Thermo Scientific, Wilmington, DE, USA). Complementary DNA was synthesized using High Capacity RNA-to-cDNA Kit based on manufacturer’s protocol (Foster City, CA, USA). The expression of exon 54 (NM_022124) was checked on CFX96 Real Time PCR detection system (Bio-Rad, Hercules, CA, USA) using Syber Premix Ex Taq at 2× (Tli RNaseH Plus, Japan). Primers sequences (listed in Table 1) were carefully designed to amplify relevant transcripts without genomic DNA contamination. Validation of amplification specificity and accuracy was confirmed through the presence of a single peak on melt curve at expected Tm and also through the generation of the correct amplicon size on agarose gel electrophoresis.

**Table 1:** *CDH23* primers for RT-PCR analysis.

Primer-pairs	Primers	Position	PCR product size (pb)	Melting temperature (°C)
1	F1 5′-TACGCCACGGACAAGGAT-3′	Ex54	286	89.5
	R 5′-GGGGGCTGCCTTTTGGAG-3′	Ex55-56		
2	F2 5′-TACAGCCTCATCTTGGTGG-3′	Ex54-Ex55	133	89.0
	R 5′-GGGGGCTGCCTTTTGGAG-3′	Ex55-Ex56		

## Results

**Figure 1:**
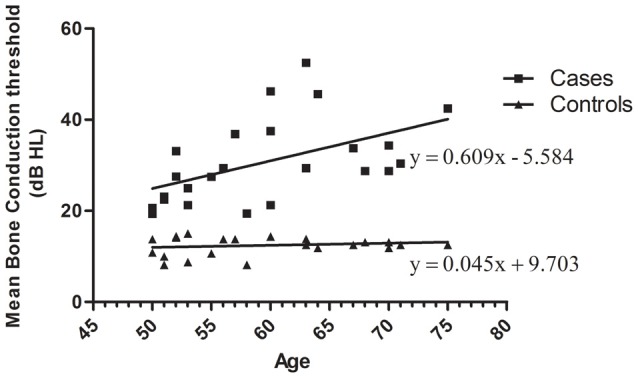
Distribution of mean bone conduction threshold as a function of age in age-related hearing impairment (ARHI) cases and healthy controls, generated using GraphPad Prism 5.0. Means of the right and left ear hearing thresholds at 500, 1000, 2000 and 4000 Hz for bone conduction in patients were plotted as a function against subjects’ age ranging from 50 to 75 years. Hearing loss thresholds are expressed in decibels (dB). Linear regression equation and line were added for each data series of cases and controls.

Presbycusis and healthy groups were carefully evaluated in terms of age match. Pure tone audiometry for air and bone conduction thresholds show no air-bone gap. The distribution of mean bone conduction threshold for each participant as function of age is plotted in Figure 1. The distribution of mean bone conduction threshold for healthy controls do not change significantly as function of the age ranging from 50 to 75 years. Whereas, for ARHI cases, the mean bone conduction threshold increases gradually with age as indicated from the linear trend line *y* = 0.609x − 5.584.

**Figure 2:**
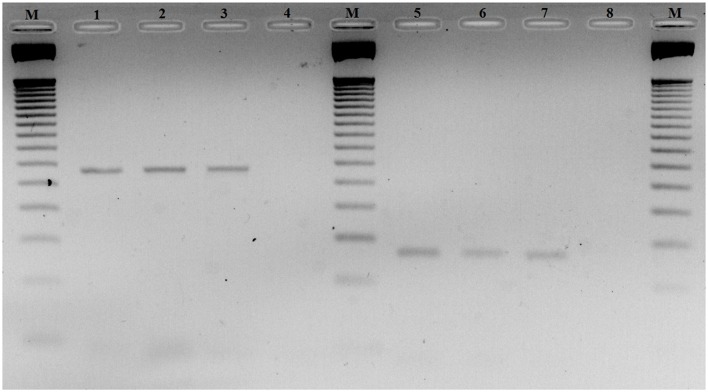
Agarose gel electrophoresis of RT-PCR products of exon 54 Cadherin 23 (*CDH23*) gene (NM_022124). Lanes M: 50-pb DNA molecular weight markers. Lanes 1, 2 and 3: RT-PCR products using primer-pairs 1 with different ages 60, 64 and 70 years old, respectively. Lanes 5, 6 and 7: RT-PCR products using primer-pairs 2 with different ages 60, 64 and 70 years old, respectively. Lanes 4 and 8: negative controls primer-pairs 1 and 2, respectively.

To investigate *CDH23* epigenetic modifications in ARHI, we screened for CpG-rich region within this gene in both ARHI cases and healthy controls. An intragenic CpG island at genomic position chr10:73, 565, 570-73, 565, 827 (GRCh37/hg19) overlapping with the junction exon 54—intron 54 (NM 022124) was selected. This region was chosen based on the high conservation score with vertebrate multiz alignment and conservation for 100 species in addition to genomic evolutionary rate profiling for mammalian alignments (Table 2). This CpG island was found to be conserved between human and mouse species genomes with 89.7% of identity. The conserved region of mouse *Cdh23* gene is localized on chr10:60,312,545-60,312,660 (GRCm38/mm10) at exon 55 for both isoforms (NM_023370) and (NM_001252635). Mouse *Cdh23* gene contains 69 exons encoding two *Cdh23* transcripts differing only by exon 68 splicing (Bork et al., 2001; Di Palma et al., 2001). Notably, these two isoforms including exon 55 are expressed in the inner ear tissues (Siemens et al., 2004; Lagziel et al., 2005; Xu et al., 2008). Moreover, RT-PCR melt curve results for peripheral blood human samples proved the amplification of the correct products of the exon 54 (NM 022124) while agarose gel electrophoresis confirmed the amplification of the expected amplicons sizes (Figure 2).

**Table 2:** Characteristic and evidence for conservation of intragenic Cadherin 23 (*CDH23*) CpG island in the human (GRCh37/hg19) and other species genomes, generated by UCSC genome Browser.

Chromosome: position	CpG number	Length	GC number	% CpG	% C or G	Obs/Exp CpG^*^	Mean phyloP100 wayAll	GERP Score	DNaseI hypersensitivity cluster^#^
chr10:73156164-73157954	CpG:171	1790	1121	19.1	62.6	0.97	0.392	0.553	69
chr10:73533091-73533492	CpG:49	401	299	24.4	74.6	0.88	0.123	0.103	13
chr10:73565570-73565827	CpG:26	258	173	20.2	67.1	0.91	2.942	2.14	58

^*^Ratio of observed to expected CpG; ^#^DNaseI Hypersensitivity Clusters in 125 cell types.

To determine methylation patterns for human *CDH23* CpG island, we carried out qMSP in a cohort of ARHI patients and healthy subjects of matched age. After bisulfite conversion of selected DNA samples, we performed amplification in the CpG island region as explained in Subjects and methods section. Different CpG methylation levels between ARHI patients and controls were evaluated applying the ΔΔCq method using the *TSH2B* as a reference gene. Methylated DNA quantification revealed 3.27-fold increase in methylation levels at common CpG island of *CDH23* in blood samples from women with presbycusis as compared to controls.

**Figure 3:**
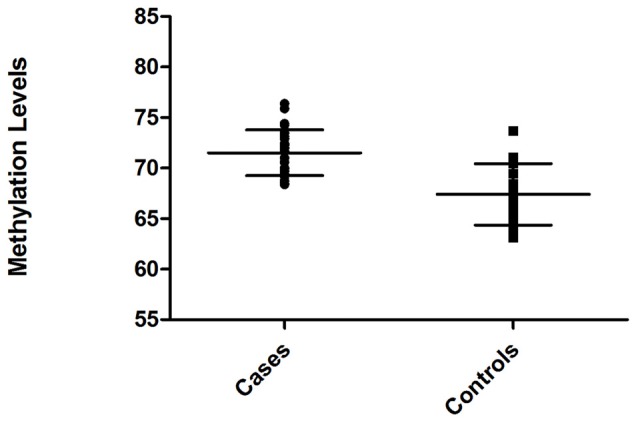
Distribution of methylation levels of intragenic *CDH23* CpG island for ARHI patients (cases) and healthy controls (Controls), generated using GraphPad Prism 5.0.

Relative level of DNA methylation was about 71.52 ± 0.45 for ARHI group, and 67.40 ± 0.61 for control group (Figure 3). Using unpaired *t*-test, this relative level of methylation was found to be significantly different (*p* < 0.0001) between the two groups. The difference in the relative level of DNA methylation between ARHI and control groups resulted in hypermethylation changes of 4.12 ± 0.76 value in ARHI patients as compared to healthy controls with a 95% CI range of 2.59–5.64.

Moreover, correlation analysis was assessed between the DNA methylation levels of *CDH23* gene for ARHI and control groups. For DNA methylation levels and hearing impairment values of ARHI and control groups, Fisher’s exact test resulted in statistically significant correlation with *p*-value of 0.036. The strength of correlation was determined with a relative risk of about 1.85 through the 95% CI [1.04–3.30]. The DNA methylation level of *CDH23* was significantly related to an increase of ARHI risk with OR of 2.22 and a 95% CI range of [1.07–4.60].

## Discussion

Presbycusis is one of the major health problems affecting elderly people, with an expanding incidence as the population age increases. It is estimated that the number of people suffering from hearing impairment will increase considerably to reach over 900 million people by 2050 (WHO, March 2018). Although several chromosomal loci and mutations have been determined in non-syndromic hearing impairment patients, many cases remain undiagnosed especially those affected with presbycusis. It develops in a multistep manner which is most likely a multifactorial process. The difficulty to identify ARHI genes could suggest the fact that it is a complex genetic disorder resulting from accumulation of several mutations in different interacting genes with lifelong cumulative effects. Environmental factors such as cumulative noise or ototoxic drugs cause sensorineural cell damage (Yang et al., 2015). Hypertension and diabetes are also reported to be major risk factors for ARHI disorder (Oron et al., 2014). Furthermore, genetic predisposition factors represent a significant scope for hearing impairment with age increase (Kvestad et al., 2012). On the other hand, progressive phenotype implies that epigenetic mechanisms could be involved in this process. Epigenetic regulation has been involved in various types of age-related human diseases (García-Giménez et al., 2012). Given the fact that they play an important role in cell development and differentiation, epigenetics are expected to significantly contribute to HI sensory disorders (Friedman and Avraham, 2009). DNA methylation, as a crucial epigenetic regulatory mechanism, is sensitive to the interaction between genetic and environmental factors. It may provide a link between the environment (Fraga et al., 2005) and genetic background (Bell et al., 2011).

In the present study, we performed a case-control comparison of intragenic *CDH23* methylation levels in peripheral blood samples. Our study suggests that there is an increased risk for ARHI through DNA methylation changes in *CDH23* gene. Obviously, DNA methylation profiles are likely to be dependent from specific conditions and to be variable from one cell-type to another. However, it was reported for instance that age effects on DNA methylation levels are well maintained between human brain and blood tissue (Horvath et al., 2012). Due to difficulty in accessing human cochlear tissue, we opted to use peripheral blood cells as propitious surrogate as reported in other disorders in which tissues are inaccessible (Mohr and Liew, 2007). Nevertheless, it is still an interesting approach with more practical convenience and significance for the discovery of potential and systemic biomarkers for presbycusis.

Empirically, DNA methylation occurring at the cytosine nucleotides in the CpG islands that are mainly located at promoter region controls gene expression (Larsen et al., 1992; Cheng et al., 2014). In fact, it was well proven that hyper-methylation of promoters correlates with a decrease or even silencing of gene expression (Suzuki and Bird, 2008). Interestingly, methylome studies showed a high density of intragenic DNA methylation compared to promoter regions (Cokus et al., 2008). Moreover, DNA methylation within body genes was found to interfere with transcript elongation in *Arabidopsis thaliana* instead of causing gene expression variation or silencing (Zilberman et al., 2007). These findings in turn raise more interests in investigating intragenic DNA methylation implications in human diseases. Here, we report for the first time DNA hypermethylation changes occurring in exon-intron junction of *CDH23* gene. It has been reported that exons were more highly sensitive to methylation than introns, and changeover in the methylation levels occurring in exon-intron junctions were considered to be important for splicing regulation (Laurent et al., 2010; Gelfman et al., 2013). *CDH23* encodes nine different isoforms (GRCh37/hg19). Transcript variant NM_022124 (isoform 1) represents the longest transcript with 68 total exons of which 67 are coding (GRCh37/hg19). Isoforms 2, 3, 4 and 5 differ in the 3’-untranslated and 3’-coding regions compared to variant 1. They are shorter than variant 1 with different C-termini. Compared to variant 1, isoforms 6 (NM_001171933.1) and 7 (NM_001171934.1) differ in the 5’-untranslated region as they lack a section of the 5’-coding region and begin translation at a downstream start codon. Also, isoform 7 lacks an in-frame exon in the 3’-coding region. Finally, isoforms 8 (NM_001171935) and 9 (NM_001171936) include only 5 and 4 coding exons, respectively. The evaluated CpG region was only common to transcript variants corresponding to isoforms 1, 6 and 7. It occurs at an exon-intron junction, which corresponds to exon-intron 54 for isoform 1 and exon-intron 9 for both isoforms 6 and 7. The comprehensive transcriptomic study (Schrauwen et al., 2016) reported *CDH23* expression levels for cochlear isoforms in elder controls. Isoform 1 (NM_022124) was the most abundant in cochlear tissues and this correlates with our findings in blood cells.

DNA methylation changes at this CpG region may cause in frame skipping of 192 bp exonic region that encodes a peptide of 64 amino acids (Glu2625-Ser2688) located at the extracellular region. DNA methylation could inhibit binding of post-transcription regulatory factors that interact with pre-mRNA to regulate exons splicing (Lev Maor et al., 2015). One in-frame and four missense variations in exon 54 (NM_022124) of *CDH23* gene are predicted as pathogenic and likely pathogenic, respectively, to cause hearing loss according to Deafness Variation Database^2^. Thus, the possible alternative skipping of exon 54 will affect *CDH23* protein function which plays a key role in hearing process. Likewise, mouse *Cdh23*^753A^ allele, underlying *Ahl* phenotype, results in-frame exon-skipping (Johnson et al., 2017). The skipped exon 7 encodes a peptide of 43 amino acids that is also located at the extracellular amino-terminus, which is in the homodimerization site and part of the 2nd and 3rd ectodomains of Cadherin 23 (Noben-Trauth et al., 2003). In-frame exon-skipping of the* Cdh23* is likely to cause partial function defect that could progressively lead to hair cell degeneration.

Considering the relevance of *CDH23* biomarker as a congenital hearing impairment gene and a good candidate gene for ARHI, risk associated studies for this particular biomarker should be replicated in large and different elderly populations. Moreover, similar studies on DNA methylation changes in other known candidate genes of ARHI should be undertaken.

## Conclusion

This study provides more evidence in respect to the identification of new epigenetic ARHI biomarkers with a guidance value to better understand the pathogenicity of ARHI complex disease. We used a non-invasive approach to compare *CDH23* methylation levels in ARHI patients against age-matched healthy subjects. Our study demonstrates the involvement of *CDH23* hypermethylation in ARHI disorder. Further investigations of DNA methylation in *CDH23* and other candidate genes would certainly assist in understanding complex pathogenic mechanisms involved in ARHI.

## Author Contributions

SM conceived and designed this study and had contributions to all its stages. AB designed and performed experiments, analyzed data and wrote the article. IS and SL collected and evaluated DNA samples. AC and AAG ascertained patients and obtained clinical data. AAG and MG critically revised the manuscript.

## Conflict of Interest Statement

The authors declare that the research was conducted in the absence of any commercial or financial relationships that could be construed as a potential conflict of interest.

## Footnotes

^1^http://www.who.int/mediacentre/factsheets/fs300/en/

^2^http://deafnessvariationdatabase.org/

## References

[B1] Bell J. T. Pai A. A. Pickrell J. K. Gaffney D. J. Pique-Regi R. Degner J. F.. (2011). DNA methylation patterns associate with genetic and gene expression variation in HapMap cell lines. Genome Biol. 12:R10. 10.1186/gb-2011-12-1-r10 21251332 PMC3091299

[B2] Bork J. M. Peters L. M. Riazuddin S. Bernstein S. L. Ahmed Z. M. Ness S. L.. (2001). Usher syndrome 1D and nonsyndromic autosomal recessive deafness DFNB12 are caused by allelic mutations of the novel cadherin-like gene CDH23. Am. J. Hum. Genet. 68, 26–37. 10.1086/316954 11090341 PMC1234923

[B3] Bouzid A. Smeti I. Dhouib L. Roche M. Achour I. Khalfallah A.. (2018). Down-expression of P2RX2, KCNQ5, ERBB3 and SOCS3 through DNA hypermethylation in elderly women with presbycusis. Biomarkers 23, 347–356. 10.1080/1354750x.2018.1427795 29325454

[B4] Bustin S. A. Benes V. Garson J. A. Hellemans J. Huggett J. Kubista M.. (2009). The MIQE guidelines: minimum information for publication of quantitative real-time PCR experiments. Clin. Chem. 55, 611–622. 10.1373/clinchem.2008.112797 19246619

[B5] Cheng J. Tang L. Hong Q. Ye H. Xu X. Xu L.. (2014). Investigation into the promoter DNA methylation of three genes (CAMK1D, CRY2 and CALM2) in the peripheral blood of patients with type 2 diabetes. Exp. Ther. Med. 8, 579–584. 10.3892/etm.2014.1766 25009623 PMC4079401

[B6] Cohen N. M. Kenigsberg E. Tanay A. (2011). Primate CpG islands are maintained by heterogeneous evolutionary regimes involving minimal selection. Cell 145, 773–786. 10.1016/j.cell.2011.04.024 21620139

[B7] Cokus S. J. Feng S. Zhang X. Chen Z. Merriman B. Haudenschild C. D.. (2008). Shotgun bisulphite sequencing of the Arabidopsis genome reveals DNA methylation patterning. Nature 452, 215–219. 10.1038/nature06745 18278030 PMC2377394

[B8] Davydov E. V. Goode D. L. Sirota M. Cooper G. M. Sidow A. Batzoglou S.. (2010). Identifying a high fraction of the human genome to be under selective constraint using GERP++. PLoS Comput. Biol. 6:e1001025. 10.1371/journal.pcbi.1001025 21152010 PMC2996323

[B9] Di Palma F. Pellegrino R. Noben-Trauth K. (2001). Genomic structure, alternative splice forms and normal and mutant alleles of cadherin 23 (Cdh23). Gene 281, 31–41. 10.1016/s0378-1119(01)00761-2 11750125

[B10] Fraga M. F. Ballestar E. Paz M. F. Ropero S. Setien F. Ballestar M. L.. (2005). Epigenetic differences arise during the lifetime of monozygotic twins. Proc. Natl. Acad. Sci. U S A 102, 10604–10609. 10.1073/pnas.0500398102 16009939 PMC1174919

[B11] Friedman L. M. Avraham K. B. (2009). MicroRNAs and epigenetic regulation in the mammalian inner ear: implications for deafness. Mamm. Genome 20, 581–603. 10.1007/s00335-009-9230-5 19876605

[B12] García-Giménez J. L. Sanchis-Gomar F. Lippi G. Mena S. Ivars D. Gomez-Cabrera M. C.. (2012). Epigenetic biomarkers: a new perspective in laboratory diagnostics. Clin. Chim. Acta 413, 1576–1582. 10.1016/j.cca.2012.05.021 22664147

[B13] Gelfman S. Cohen N. Yearim A. Ast G. (2013). DNA-methylation effect on cotranscriptional splicing is dependent on GC architecture of the exon-intron structure. Genome Res. 23, 789–799. 10.1101/gr.143503.112 23502848 PMC3638135

[B14] Horvath S. Zhang Y. Langfelder P. Kahn R. S. Boks M. P. M. van Eijk K.. (2012). Aging effects on DNA methylation modules in human brain and blood tissue. Genome Biol. 13:R97. 10.1186/gb-2012-13-10-r97 23034122 PMC4053733

[B15] Hwang J.-H. Liu K. S. Wu C.-C. Liu T.-C. (2012). Association of Cadherin23 single nucleotide polymorphism with age-related hearing impairment in Han Chinese. Otolaryngol. Head Neck Surg. 147, 531–534. 10.1177/0194599812446904 22581638

[B16] Johnson K. R. Tian C. Gagnon L. H. Jiang H. Ding D. Salvi R.. (2017). Effects of *Cdh23* single nucleotide substitutions on age-related hearing loss in C57BL/6 and 129S1/Sv mice and comparisons with congenic strains. Sci. Rep. 7:44450. 10.1038/srep44450 28287619 PMC5347380

[B17] Johnson K. R. Zheng Q. Y. Erway L. C. (2000). A major gene affecting age-related hearing loss is common to at least ten inbred strains of mice. Genomics 70, 171–180. 10.1006/geno.2000.6377 11112345

[B18] Kvestad E. Czajkowski N. Krog N. H. Engdahl B. Tambs K. (2012). Heritability of hearing loss. Epidemiology 23, 328–331. 10.1097/ede.0b013e318245996e 22249243

[B19] Lagziel A. Ahmed Z. M. Schultz J. M. Morell R. J. Belyantseva I. A. Friedman T. B.. (2005). Spatiotemporal pattern and isoforms of cadherin 23 in wild type and waltzer mice during inner ear hair cell development. Dev. Biol. 280, 295–306. 10.1016/j.ydbio.2005.01.015 15882574

[B20] Larsen F. Gundersen G. Lopez R. Prydz H. (1992). CpG islands as gene markers in the human genome. Genomics 13, 1095–1107. 10.1016/0888-7543(92)90024-m 1505946

[B21] Laurent L. Wong E. Li G. Huynh T. Tsirigos A. Ong C. T.. (2010). Dynamic changes in the human methylome during differentiation. Genome Res. 20, 320–331. 10.1101/gr.101907.109 20133333 PMC2840979

[B22] Lev Maor G. Yearim A. Ast G. (2015). The alternative role of DNA methylation in splicing regulation. Trends Genet. 31, 274–280. 10.1016/j.tig.2015.03.002 25837375

[B23] McHugh R. K. Friedman R. A. (2006). Genetics of hearing loss: Allelism and modifier genes produce a phenotypic continuum. Anat. Rec. A Discov. Mol. Cell. Evol. Biol. 288, 370–381. 10.1002/ar.a.20297 16550584

[B24] Mohr S. Liew C.-C. (2007). The peripheral-blood transcriptome: new insights into disease and risk assessment. Trends Mol. Med. 13, 422–432. 10.1016/j.molmed.2007.08.003 17919976

[B25] Noben-Trauth K. Zheng Q. Y. Johnson K. R. (2003). Association of cadherin 23 with polygenic inheritance and genetic modification of sensorineural hearing loss. Nat. Genet. 35, 21–23. 10.1038/ng1226 12910270 PMC2864026

[B26] Oron Y. Elgart K. Marom T. Roth Y. (2014). Cardiovascular risk factors as causes for hearing impairment. Audiol. Neurootol. 19, 256–260. 10.1159/000363215 25073427

[B27] Petit C. Levilliers J. Hardelin J.-P. (2001). Molecular genetics of hearing loss. Annu. Rev. Genet. 35, 589–645. 10.1146/annurev.genet.35.102401.091224 11700295

[B28] Pickles J. O. Comis S. D. Osborne M. P. (1984). Cross-links between stereocilia in the guinea pig organ of Corti and their possible relation to sensory transduction. Hear. Res. 15, 103–112. 10.1016/0378-5955(84)90041-8 6436216

[B29] Provenzano M. J. Domann F. E. (2007). A role for epigenetics in hearing: establishment and maintenance of auditory specific gene expression patterns. Hear. Res. 233, 1–13. 10.1016/j.heares.2007.07.002 17723285 PMC2994318

[B30] Schmittgen T. D. Livak K. J. (2008). Analyzing real-time PCR data by the comparative C_T_ method. Nat. Protoc. 3, 1101–1108. 10.1038/nprot.2008.73 18546601

[B31] Schrauwen I. Hasin-Brumshtein Y. Corneveaux J. J. Ohmen J. White C. Allen A. N.. (2016). A comprehensive catalogue of the coding and non-coding transcripts of the human inner ear. Hear. Res. 333, 266–274. 10.1016/j.heares.2015.08.013 26341477 PMC4775449

[B32] Siemens J. Lillo C. Dumont R. A. Reynolds A. Williams D. S. Gillespie P. G.. (2004). Cadherin 23 is a component of the tip link in hair-cell stereocilia. Nature 428, 950–955. 10.1038/nature02483 15057245

[B34] Siepel A. Bejerano G. Pedersen J. S. Hinrichs A. S. Hou M. Rosenbloom K.. (2005). Evolutionarily conserved elements in vertebrate, insect, worm, and yeast genomes. Genome Res. 15, 1034–1050. 10.1101/gr.3715005 16024819 PMC1182216

[B35] Suzuki M. M. Bird A. (2008). DNA methylation landscapes: provocative insights from epigenomics. Nat. Rev. Genet. 9, 465–476. 10.1038/nrg2341 18463664

[B36] Tu N. C. Friedman R. A. (2018). Age-related hearing loss: unraveling the pieces. Laryngoscope Investig. Otolaryngol. 21, 68–72. 10.1002/lio2.134 29721536 PMC5915820

[B37] Wu X. Wang Y. Sun Y. Chen S. Zhang S. Shen L.. (2014). Reduced expression of Connexin26 and its DNA promoter hypermethylation in the inner ear of mimetic aging rats induced by d-galactose. Biochem. Biophys. Res. Commun. 452, 340–346. 10.1016/j.bbrc.2014.08.063 25159847

[B39] Xu Z. Peng A. W. Oshima K. Heller S. (2008). MAGI-1, A candidate stereociliary scaffolding protein, associates with the tip-link component cadherin (23). J. Neurosci. 28, 11269–11276. 10.1523/JNEUROSCI.3833-08.2008 18971469 PMC2596868

[B38] Xu J. Zheng J. Shen W. Ma L. Zhao M. Wang X.. (2017). Elevated SLC26A4 gene promoter methylation is associated with the risk of presbycusis in men. Mol. Med. Rep. 16, 347–352. 10.3892/mmr.2017.6565 28498466

[B40] Yang C.-H. Schrepfer T. Schacht J. (2015). Age-related hearing impairment and the triad of acquired hearing loss. Front. Cell. Neurosci. 9:276. 10.3389/fncel.2015.00276 26283913 PMC4515558

[B41] Zilberman D. Gehring M. Tran R. K. Ballinger T. Henikoff S. (2007). Genome-wide analysis of Arabidopsis thaliana DNA methylation uncovers an interdependence between methylation and transcription. Nat. Genet. 39, 61–69. 10.1038/ng1929 17128275

